# Constant–Murley Score: systematic review and standardized evaluation in different shoulder pathologies

**DOI:** 10.1007/s11136-018-1875-7

**Published:** 2018-05-10

**Authors:** Kalliopi Vrotsou, Mónica Ávila, Mónica Machón, Maider Mateo-Abad, Yolanda Pardo, Olatz Garin, Carlos Zaror, Nerea González, Antonio Escobar, Ricardo Cuéllar

**Affiliations:** 1 Unidad de Investigación de Atención Primaria-OSIS Gipuzkoa, Instituto Biodonostia, Paseo Dr. Beguiristain s/n, 20014 San Sebastián, Spain; 2 Red de Investigación en Servicios de Salud en Enfermedades Crónicas (REDISSEC), Bilbao, Spain; 3 Centro de Investigación en Cronicidad Kronikgune, Barakaldo, Spain; 4 Health Services Research Unit, IMIM (Hospital del Mar Medical Research Institute), Barcelona, Spain; 5 CIBER Epidemiología y Salud Pública, Barcelona, Spain; 6 Universitat Pompeu Fabra, Barcelona, Spain; 7 Department of Paediatric Dentistry and Orthodontics, Faculty of Dentistry, Universidad de La Frontera, Temuco, Chile; 8 Unidad de Investigación, Hospital Galdakao-Usansolo, Galdakao, Spain; 9 Unidad de investigación, Hospital Universitario Basurto, Bilbao, Spain; 10 Servicio de Traumatología y Cirugía Ortopédica, Hospital Universitario Donostia, San Sebastián, Spain; 11 Faculty of Dentistry, Universidad San Sebastian, Puerto Montt, Chile

**Keywords:** Constant–Murley score, Systematic review, Shoulder pathologies, EMPRO tool, Standardized evaluation, Psychometric properties

## Abstract

**Purpose:**

The objective of this study was to evaluate the psychometric properties of the Constant–Murley Score (CMS) in various shoulder pathologies, based on a systematic review and expert standardized evaluations.

**Methods:**

A systematic review was performed in MEDLINE and EMBASE databases. Titles and abstracts were reviewed and finally the included articles were grouped according to patients' pathologies. Two expert evaluators independently assessed the CMS properties of reliability, validity, responsiveness to change, interpretability and burden score in each group, using the EMPRO (Evaluating Measures of Patient Reported Outcomes) tool. The CMS properties were assessed per attribute and overall for each considered group. Only the concept and measurement model was assessed globally.

**Results:**

Five individual pathologies (i.e. subacromial, fractures, arthritis, instability and frozen shoulder) and two additional groups (i.e. various pathologies and healthy subjects) were considered. Overall EMPRO scores ranged from 58.6 for subacromial to 30.6 points for instability. Responsiveness to change was the only quality to obtain at least 50 points across all groups, but for frozen shoulder. Insufficient information was obtained in relation to the concept and measurement model and great variability was seen in the other evaluated attributes.

**Conclusions:**

The current evidence does not support the CMS as a gold standard in shoulder evaluation. Its use is advisable for subacromial pathology; but data are inconclusive for other shoulder conditions. Prospective studies exploring the psychometric properties of the scale, particularly for fractures, arthritis, instability and frozen shoulder are needed.

**Level of evidence:**

Systematic review.

**Electronic supplementary material:**

The online version of this article (10.1007/s11136-018-1875-7) contains supplementary material, which is available to authorized users.

## Introduction

The Constant–Murley Score (CMS) was presented in 1987 as an instrument to evaluate overall shoulder function, irrespective of diagnosis [1]. It was approved and recommended by the executive committee of the European Society for Surgery of the Shoulder and the Elbow and has been widely used as an assessment method ever since [2–4].

The CMS scale assesses four aspects related to shoulder pathology; two subjective: pain and activities of daily living (ADL) and two objective: range of motion (ROM) and strength. The subjective components can receive up to 35 points and the objective 65, resulting in a possible maximum total score of 100 points (best function). Pain and ADL are answered by the patient; ROM and strength require a physical evaluation and are answered by the orthopaedic surgeon or the physiotherapist [1].

Despite its wide acceptance and frequent use, certain concerns related to the suitability of the CMS scale have been raised over the years. A number of publications mention lack of information, as far as the methodology used during its development process, item selection criteria, score distribution, reliability and validity are concerned [2, 3, 5, 6]. Others have questioned its application to certain shoulder pathologies [5, 7, 8]; differences according to age and sex have been observed [9, 10] and lack of standardization in measuring the strength component has been criticized [11, 12].

In an attempt to clarify certain aspects related to its administration, the original author published an article with modifications and guidelines for the instrument´s use in 2008 [13]. A visual analog scale (VAS) was suggested for the pain item, and part of the ADL questions and specific instructions on how to evaluate the strength component were presented. It was also stated that the CMS is not valid for evaluating episodic severe pain, as in dislocation. Finally, a score modification, adjusting for age and sex was proposed [13].

The psychometric properties of the CMS questionnaire have been the subject of literature reviews [3, 4], general systematic reviews [14] and reviews on specific shoulder pathologies [15, 16]. However, up to date, no standardized evaluation of its properties in various shoulder diagnoses has been presented.

The evaluating measures of patient reported outcomes (EMPRO) tool was created for evaluating the psychometric properties of patient reported outcomes (PRO) [17]. This tool is composed of a broad spectrum of questions and specific recommendations on how each property should be assessed. It requires the involvement of expert evaluators and offers standardized and comparable results. It assesses the concept and measurement model of a scale as well as the attributes of reliability, validity and responsiveness to change, among others; and it has been previously used in the evaluation of different PRO scales [18–20].

The purpose of the current study was to perform a systematic literature review and a standardized evaluation of the CMS properties. The evidence was grouped according to the type of shoulder diagnosis. Subacromial, fractures, arthritis, instability and frozen shoulder pathologies were assessed, while data on various pathologies and healthy subjects were also evaluated. The current results will offer clinicians and researchers more insight on the CMS psychometric properties, allowing for the latter to be compared between different diagnostic groups. To the best of our knowledge, it is the first time that a CMS evaluation with these characteristics is performed.

## Materials and methods

### Literature review

Systematic searches were conducted in MEDLINE and EMBASE databases for the period between January 1st 1986 and May 2nd 2014. For specific strategies see Online Appendix 1.

Articles presenting information on the development process, the psychometric properties and the administration of the CMS tool were eligible for inclusion. Articles written in English, Spanish, French, German and Italian were included in the evaluation stage. Opinion letters, congress abstracts, study protocols, case studies, articles on animal and cadaveric studies presenting information on surgical or other techniques applicable to shoulder pathologies were excluded.

Titles, abstracts and full texts were independently reviewed by two investigators (KV & MA) in a three-step process. A third researcher (YP) was appointed to resolve possible discrepancies if needed. In order to complete the search, the reference lists of all finally selected articles were also hand searched. General shoulder review articles were not given to the evaluators, but were read and their references hand searched by the previous two authors. Review articles on specific shoulder pathologies were not evaluated per se, but were given to the evaluators for consideration and possible identification of further references on relevant data.

Patient pathologies of all included articles were noted and were subsequently grouped according to their characteristics. The grouping criteria were established by one of the co-authors (RC: orthopaedic surgeon experienced in upper extremities), considering the main shoulder pathologies, in line with the indications of the American Academy of Orthopaedic Surgeons (AAOS).

### The CMS scale

The CMS is a multi-item functional scale assessing pain, ADL, ROM and strength of the affected shoulder. Its score ranges from 0 to 100 points, representing worst and best shoulder function, respectively.

In the original publication, the pain experienced during normal activities of daily living was scored as: no pain = 15 points, mild = 10, moderate = 5 and severe = 0 points [1]. The most recent publication recommends these options to be replaced by a VAS, maintaining the 15 points score range [13].

The ADL component is assigned a maximum of 20 points and evaluates limitations in doing normal work, recreational activities, unaffected night sleep and positioning the arm up to a certain level. The first two items were originally scored as: no limitation = 4, moderate = 2 and severe = 0 points [1]. In the latest publication a VAS was suggested for both questions [13], while the score range of the other two would remain the same. Night sleep is assessed as: unaffected = 2, sometimes disturbed = 1, always disturbed = 0 points. And finally arm positioning: up to waist = 2, xiphoid = 4, neck = 6, head = 8, above head = 10 points.

The ROM part evaluates four active ranges of motion, receiving 10 points each, i.e. pain-free forward and lateral elevation, external and internal rotation. Elevation degrees are measured with a goniometer in a seated position and scores range from: 0°–30° = 0 to 151°–180° = 10 points. External rotation is based on five unassisted hand manoeuvers, assigned 2 points each: hand behind head with elbow forward, hand behind head with elbow back, hand on top of head with elbow forward, hand on top of head with elbow back and full elevation. Internal rotation was initially measured with the dorsum of the hand pointing to certain parts of the body, but in the most recent publication, the thumb was suggested as a pointer to the following anatomic landmarks: lateral thigh = 0, buttock = 2, lumbosacral junction = 4, waist = 6, 12th dorsal vertebra = 8 and interscapular region = 10 points.

The strength component is given 25 points. Originally, the use of an unsecured cable tensiometer or spring balance was instructed and scoring was based on the number of pounds of pull that a subject could resist, in up to a maximum of 90° of abduction [1]. In the updated recommendations, this is done at 90° of abduction, with the hand facing downward, using either a dynamometer or a defined spring balance technique. The maximum value of three consecutive repetitions should be used. When desired abduction cannot be reached, then the subject is given 0 points [13].

Given the importance that age and sex have in the functional capacity of the shoulder, an alternative CMS scoring, adjusting for these two variables, was also proposed. Based on values derived by 900 healthy subjects, the relative CMS is calculated as the original CMS divided by the respective age and sex-matched healthy values [13].

### The EMPRO tool

The EMPRO is a standardized scale, designed to evaluate the psychometric properties of PRO questionnaires, based on published evidence [17]. It is composed of 39 items divided into 8 attributes: concept and measurement model, reliability, validity, responsiveness to change, interpretability, respondent and administrative burden, alternative modes of administration and finally cross-cultural and linguistic adaptations. Each item is accompanied by specific instructions, and rated on a 4-point Likert scale from 1 (strongly disagree) to 4 (strongly agree) and include a “no information” option. Five items have an additional “not applicable” option. The EMPRO is a reliable and valid tool and has been used in the evaluation of condition specific and generic PRO instruments [17, 19–22]. Eleven shoulder PRO scales have also been evaluated with this tool [18].

### Standardized evaluations

The articles corresponding to each diagnostic group were rated independently, via EMPRO, by 2 evaluators with expertise in PRO. Most evaluators belonged to the EMPRO development working group or had undergone an EMPRO training course. All evaluators reviewed the corresponding full text articles, filled in the assessment tool and were subsequently given access to the evaluation of their pair. Discrepancies were discussed and a final consensus was reached in all cases.

### EMPRO scores

An attribute and an overall score were derived per pathology. Attribute scores were the response mean of all replies when at least 50% of the attribute items were rated; otherwise no score was given. Items with “no information” were assigned 1 point (lowest possible), while “not applicable” items were assigned the mean value of the rest of the attribute items, excluding the “no information” ones. Mean responses were linearly transformed to a 100-point scale, with higher values suggesting better properties; scores of 50 or more points are considered to be acceptable [18]. Two sub-scores are estimated for the attributes of reliability (i.e. internal consistency and reproducibility) and burden (i.e. respondents and administrative burden), with the highest among the two being the attribute´s global score. The burden scores are presented separately and are not affecting any further calculations. The overall EMPRO score was based on the rating of 5 attributes: concept and measurement model, reliability, validity, responsiveness and interpretability. This score was calculated if at least 3 of those 5 attributes had a rating and attributes with insufficient information were given 0 points. The rating algorithm was run in SPSS version 23 (SPSS, Chicago, IL, USA).

For the needs of this study, the attribute of concept and measurement model was evaluated only once, by two of the authors (KV & MA), also participating in the evaluation process. It was not deemed necessary for all reviewers to repeat this evaluation, given that the same published information would have to be evaluated by all. The score of this attribute entered in the final EMPRO score of all considered pathology groups.

It was hypothesized that respondent burden would vary according to pathology; while aspects like “time required” and “training and expertise needed”, assessed as part of the administrative burden, may also depend upon it. For these reasons, the two burden attributes were evaluated per pathology group. Likewise, the alternative forms of administration attribute, were also evaluated per pathology. None of these three attributes is included in the final EMPRO scores.

## Results

**Fig. 1:**
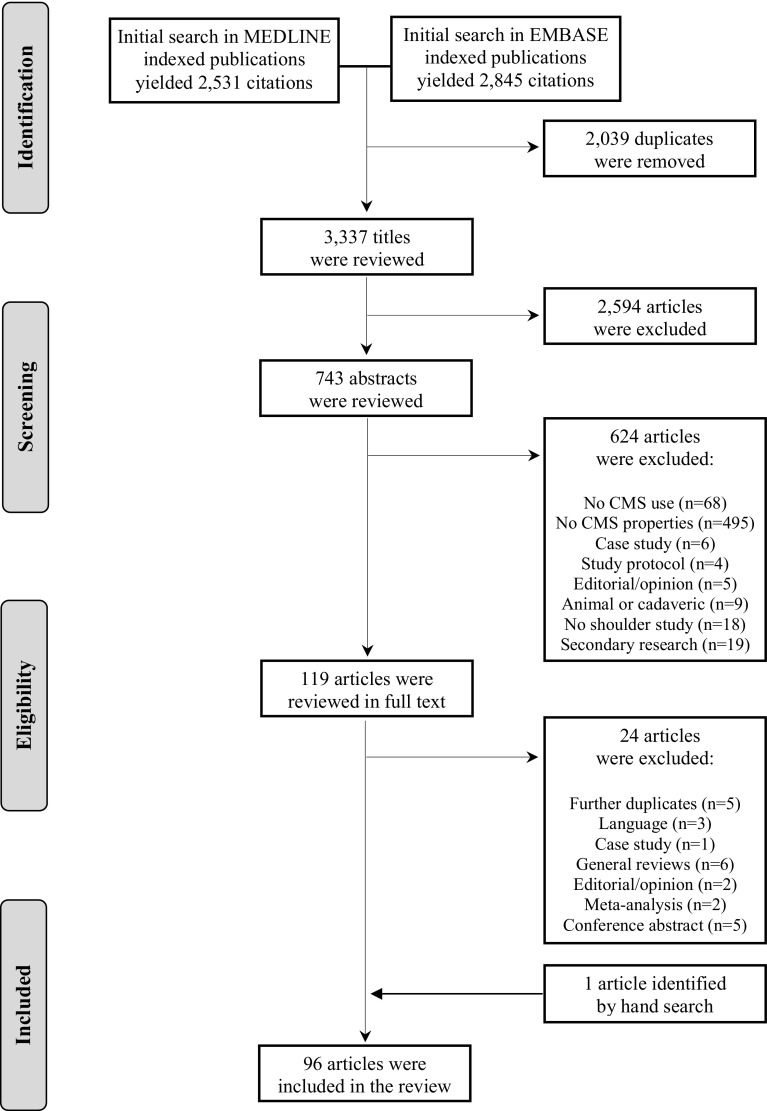
PRISMA flowchart with numbers of included and excluded articles at each step of the systematic literature review

The systematic literature search identified 3337 unique titles, of those 2594 were excluded, for not being related to the studied topic. A total of 743 abstracts were reviewed, of which 624 were excluded, mainly for not mentioning CMS use (68) or not reporting data on CMS properties (495). The rest were excluded for being secondary research articles, case studies, study protocols, commentaries, animal and cadaveric and no shoulder related studies. Finally, at the full text revision phase, 24 articles were additionally excluded for not fulfilling the inclusion criteria. One article was identified by hand search. Thus, a total of 96 full text articles were considered at the EMPRO evaluation phase (Fig. 1).

The included articles were subsequently divided into five individual pathology groups, named: subacromial pathology, fractures, arthritis, instability and frozen shoulder. Studies presenting data on heterogeneous shoulder pathologies (various pathologies) and studies on healthy subjects were also evaluated. Information on the exact pathologies considered and the number of finally included articles per group is presented in Table 1.

**Table 1:** Included pathologies and number of finally evaluated articles per group

	Included pathologies	No. articles
Individual pathologies		
Subacromial pathology	Impingement syndrome rotator cuff deficiencies; bursitis tendinitis, tendinosis of the shoulder calcific tendinitis of the shoulder	37
Fractures	Proximal humeral fractures	7
Arthritis	Glenohumeral osteoarthritis; rheumatoid arthritis; degenerative shoulder joint disease; avascular necrosis of the humeral head	6
Instability	Traumatic or non-traumatic shoulder instability; recurrent luxation recurrent dislocation	5
Frozen shoulder	Frozen shoulder; adhesive capsulitis	1
Additional groups		
Various pathologies	Various pathologies; shoulder pain	29
Healthy subjects	No shoulder pathology; healthy individuals	9
Concept & measurement model^a^		2
Total		96

^a^The concept and measurement model was evaluated globally for the CMS scale

Each pair of evaluators reviewed between 1 (i.e. frozen shoulder) and 37 (i.e. subacromial pathology) published articles. Articles presenting elaborate data on more than one pathologies were additionally given to the corresponding pathology group evaluators. The subacromial pathology evaluators also assessed the concept and measurement model attribute based on the two publications written by the original CMS author. The list of all considered publications is presented in Online Appendix 2.

The total EMPRO scores of the individual pathology groups ranged from a maximum of 58.6 points for subacromial pathology to a minimum of 30.6 points for instability (Table 2). The subacromial group was the only one to surpass the threshold of 50 total points. Fractures and arthritis obtained 43.5 and 41.7 points, respectively. Various pathologies and healthy subjects were assigned 49.3 and 37.9 points each. Information on CMS properties in frozen shoulder was insufficient. For this reason, neither attribute (but the concept and measurement model), nor total EMPRO scores were derived.

**Table 2:** Item, attribute and total EMPRO scores for all considered pathology groups

Attribute	Individual pathology groups					Additional groups	
	Subacromial pathology	Fractures	Arthritis	Instability	Frozen shoulder	Various pathologies	Healthy subjects^ǂ^
Concept and measurement model^¥^	33.3	33.3	33.3	33.3	33.3	33.3	33.3
Reliability: global score	70.8	58.3	33.3	0		54.2	62.5
Reliability: internal consistency	25					37.5	
Data collection methods described	+++	–	–	–	–	+++	–
Cronbach alpha adequate	++	–	–	–	–	+++	–
IRT estimates provided	–	–	–	–	–	–	–
Testing in different populations	–	NA	–	–	–	++	–
Reliability: reproducibility	70.8	58.3	33.3	0		54.2	62.5
Data collection methods described	++++	+++	++	+	–	+++	++++
Test–retest and time interval adequate	++++	+++	+++	+	–	++++	++++
Reproducibility coefficients adequate	++++	++++	++	+	–	+++	+++
IRT estimates provided	–	–	–	–	–	–	–
Validity	44.4	31.3	41.7	41.7		53.3	55.6
Content validity adequate	–	+	+	–	–	+	–
Construct/criterion validity adequate	+++	+++	+++	++	–	++++	+++
Sample composition described	+++	++	++++	++	–	++++	+++
Prior hypothesis stated	+++	+++	+++	+++	+	+++	+++
Rationale for criterion validity	NA	NA	NA	NA	NA	NA	NA
Tested in different populations	–	–	–	+++	–	++	++++
Responsiveness to change	83.3	50	55.6	50		55.6	
Adequacy of methods	+++	++++	+++	++++	+	+++	–
Description of estimated change magnitude	++++	+++	++++	+++	–	+++	–
Comparison of stable and unstable groups	++++	++	++	+	–	++	–
Interpretability	61.1	44.4	44.4	27.8		50	
Rationale of external criteria	++++	+++	+++	++	–	+++	++++
Description of interpretation strategies	+++	++	+++	++	–	++	–
How data should be reported stated	++	++	–	++	–	+++	–
Total EMPRO score	58.6	43.5	41.7	30.6		49.3	37.9
Burden score							
Burden I: respondent	61.1		33.3	16.7		44.4	
Skills and time needed	+++	–	+++	++	–	+++	–
Impact on respondents	++	+	++	++	–	+	++
Not suitable circumstances	++++	–	–	+	–	+++	–
Burden II: administrative	50		83.3	20.8		75.0	
Resources required	+	+++	++++	+	–	+++	–
Time required	+++	–	+++	+	–	++++	–
Training and expertise needed	++	–	+++	+	–	+++	–
Burden of score calculation	++++	–	++++	++++	–	+++	+
Alternative forms of administration						33.3	
Metric characteristics of alternative forms	–	–	–	–	–	++	–
Comparability of alternative forms	–	–	–	–	–	++	–

Scores range from strongly agree (++++) to strongly disagree (+) and no information (–), not applicable (NA). *IRT* item response theory. For all pathology groups, the overall EMPRO scores include the Concept and measurement model score of 33.3 points.

^¥^The items of concept and measurement model attribute were evaluated globally as follows: Concept of measurement stated (++++); Obtaining and combining items described (+); Rationality for dimensionality and scales (+); Involvement of target population (–); Scale variability described and adequate (+++); Level of measurement described (++); Procedures for deriving scores (++)

^ǂ^Healthy subjects score was based on four attributes, excluding Responsiveness to change

Internal consistency scores were low and calculated only for the subacromial and various pathologies groups, which obtained 25 and 37.5 points, respectively. On the other hand, reproducibility scores were noticeably higher. Over 50 points were given to subacromial and fracture groups. Arthritis was assigned 33.3 points and instability obtained the lowest possible EMPRO score of 0 points. Both various pathologies and healthy subjects had values > 50 in this attribute. Lack of item response theory (IRT) information penalized reproducibility evaluations.

Validity scores of the five individual pathology groups, oscillated between 44.4 points for subacromial diagnoses and 31.3 points for fractures, while arthritis and instability, both obtained 41.7 points. Various pathologies and healthy subjects were assigned 53.3 and 55.6 points, respectively.

Responsiveness to change was overall the best evaluated property, with all obtained scores being between 83.3 and 50 points. The highest score among the five individual pathologies was obtained by the subacromial group, followed by arthritis, fractures and instability. In addition, various pathologies obtained 55.6 points, while no score was calculated for healthy subjects. Responsiveness to change is not usually evaluated in healthy subjects, as no change is expected in this group. This property was excluded when calculating the healthy subjects total score.

As far as the attribute of interpretability was concerned, within the individual pathology groups, the highest score was 61.1 points for subacromial pathology. Instability presented the lowest value of 27.8 points and the other two groups obtained 44.4 points each. Among the additional groups, various pathologies received 50 points and no score was calculated for healthy subjects.

In relation to the respondent and administrative burden scores, the subacromial group obtained ≥ 50 in both attributes. Arthritis obtained 33.3 and 83.3 points, whereas for instability both values were < 21 points. Various pathologies reached 44.4 points for respondent and 75 points for administrative burden, while no scores were obtained for the healthy subjects group.

The attribute of alternative forms of administration was evaluated only for various pathologies, reaching 33.3 points. This evaluation was based on an article presenting a totally self-administered CMS tool. Based on a series of explicit instructions and photos, subjects are guided on how to reply the ROM and strength parts of the scale, originally designed for the clinicians [23]. The cultural adaptation attribute was not evaluated in this study. Information based on culturally adapted CMS versions was not assessed separately; it was considered part of the standardized evaluation. This approach has also been followed in previous articles [18].

## Discussion

The CMS scale has been accepted and widely used, without ever being properly validated [2, 4, 13, 14]. In the current study the psychometric properties of the CMS were assessed, in seven pathology groups, by expert evaluators using the EMPRO tool. In general, assigned scores were low. Subacromial and various pathologies obtained the best overall evaluations, but only the first group’s total EMPRO score was considered acceptable. Healthy subjects presented higher attribute scores, compared to most individual pathologies. This was due to the fact that most of the respective publications were evaluating at least one CMS property. They were thus more likely to adequately analyse and report the corresponding information. Lack of interpretability data penalized this group´s total score. In shoulder fractures, reproducibility surpassed the desired threshold and responsiveness to change was borderline, but none of the other attributes were regarded as adequate. Others have argued that the current evidence does not really support the broad CMS use in this kind of patients [15, 24]. Responsiveness to change and administrative burden were the only two attributes with acceptable estimations for arthritis. Administrating the CMS in rheumatoid arthritis patients has also been criticized; mainly due to the difficulty of an accurate strength component registration [25]. Shoulder instability can lead to luxation episodes and severe pain, reducing overall function, but these characteristics are not constantly present, which is why the CMS cannot properly assess this particular condition. This has been previously addressed and also accepted by the original CMS author [5, 13, 26, 27]. The current study corroborates this already known fact. Expert evaluations did not indicate good CMS properties for frozen shoulder. It is worth mentioning that one of the purposes of the single article assessed in that group was studying the CMS drawbacks when administered to frozen shoulder patients [7].

As far as the different psychometric properties are concerned, responsiveness to change and reliability are of major importance to the clinicians [28–30]. Instruments capable of capturing changes over time, and free of random error are of great relevance. In the current study, responsiveness to change was the best evaluated quality, with ≥ 50 points across all groups (but frozen shoulder). Among them, better evidence was obtained for subacromial pathology. Further information, especially in relation to comparing stable and unstable patients, would have been desirable in the other groups.

Reliability was overall the second best scored quality, with reproducibility being more frequently and adequately presented than internal consistency. Cronbach alphas were > 0.60, but a value of 0.37 was also seen [31]. Scarce information was surprising, as internal consistency is one of the commonest reported scale properties [28]. Given that many perceive the CMS as a gold standard, it may be that internal consistency is not of concern to them. On the other hand, it may also reflect selective reporting. A recently published study, on patients with humeral fractures, concluded insufficient evidence as far as the CMS internal consistency was concerned [32].

The available evidence supports scale reproducibility for subacromial pathology and healthy subjects. Fractures and various pathologies also scored over the established threshold but information, particularly on data collection methods, was not sufficient. A previous systematic review reported the CMS reproducibility to be acceptable in different shoulder conditions [14]. However, some of the corresponding estimations were based on Spearman´s correlation coefficients. This statistic does not capture systematic score differences and cannot be considered an appropriate reproducibility measure [33]. Intraclass correlation coefficients or the 95% limits of agreement are more adequate methods for evaluating this property [17, 34].

Validity, the degree to which an instrument measures what is supposed to measure [30], was acceptable only for various pathologies and healthy subjects. Within this attribute, content validity was the worse evaluated aspect in all groups. On the other hand prior hypothesis related to convergent and known group validity, received the same score (+++) across all shoulder diagnoses.

Interpretability, the degree to which a scale´s score can be assigned an easily understood meaning [30], was acceptable for the subacromial and on the threshold for various pathologies. The concept and measurement model attribute, highlighted that data on the CMS development are scarce. Even though the concept of measurement has been clearly stated and scale variability properly described, no information related to a target population has been presented. Rationale for item selection and scale components is insufficient, whereas the level of measurement and justification of score derivation are not properly explained. Similar observations have been made by previous authors [2–4].

Finally, great variability was observed in the burden attributes. Subacromial pathology was the only one with an acceptable respondent burden. This group, along with various pathologies and arthritis also obtained the highest scores in administrative burden.

At this point, it is relevant to mention that subacromial pathology was the most frequent shoulder condition in the various articles regarding pathologies. It is thus likely that the evaluations of this very group may have been affected by this fact.

Based on 34 articles, and performing a descriptive synthesis of the evidence, the above-mentioned work of Roy et al. concluded that the convergent validity of the scale was well established, reliability coefficient values reached acceptable benchmarks and that, with the exception of shoulder instability, the CMS had excellent responsiveness [14]. Our results support the convergent validity of the scale, with the exception of frozen shoulder, but disagree with the generalizability of the other two statements. According to the current evaluations, based on broader evidence, reliability cannot be claimed in the cases of arthritis, instability and frozen shoulder. Further responsiveness to change information, would have been desirable for all, but the subacromial pathology group.

Recently, another systematic review and standardized evaluation of various shoulder scales in rotator cuff patients, using the COSMIN checklist was published [16]. Based on 17 articles the authors concluded positive evidence for CMS reproducibility and responsiveness, indeterminate evidence for internal consistency, measurement error and criterion validity, while negative or lack of evidence was found for the rest evaluated attributes.

The administered intervention is an important factor in the evolution of any pathology [35]. The possibility of additional evaluations, considering the applied intervention, irrespective of diagnosis, was contemplated in a secondary phase of this study. However, grouping the articles anew, based on this characteristic, was very difficult to accomplish. Single studies applied different interventions for the same underlying pathologies; frequently information on intervention type and procedures was not available and commonly results were presented globally, ignoring the intervention type. For the above reasons no such evaluations were eventually performed.

Certain limitations should be addressed. After the latest recommendations [13], the modified CMS version (i.e. VAS for pain and ADL activities) or the age and sex adjusted score, has been implemented in certain publications. Most articles presenting these “updated” scores also reported the original CMS values. When this was not the case, the “updated” values were considered to be the same as the original ones. The recommended modifications are perceived as improvements of an instrument, rather than different score tools, which justifies their joint evaluation. While, due to the low number of these publications, separate evaluations would not have been possible. An additional limitation is the fact that most included articles did not explain the exact way of assessing the scale´s strength component. It is possible that studies implementing an ISOBEX dynamometer or similar, as recently recommended [13], obtained more accurate and reproducible findings. Information related to the exact CMS administration is still to be improved. The reviewers’ expertise may have introduced certain variability to the obtained results. However, EMPRO-specific instructions and consensus of all evaluations should have minimized the effect of this bias. Finally, the number of articles identified per group may have affected certain evaluations. It is important to highlight that the inclusion criteria were applied irrespective of diagnoses. Included articles were those presenting psychometric information. Grouping them by diagnosis was done a posteriori. There is a chance that better evidence could be provided if more publications were found, but the systematic literature review steps followed and the specific inclusion criteria should have reduced the possibility of excluding relevant articles. Finally, the EMPRO instrument was created for evaluating PRO and the CMS is a functional scale with a PRO component. Nonetheless, it is accepted that both instrument types should possess the same psychometric properties, which justifies the aim and approach of the current study [28, 36].

The systematic review followed by expert evaluations constitute the main strengths of this work. To the best of our knowledge our study is the first in using the EMPRO assessment tool for exploring the CMS attributes in different shoulder pathologies. The current results offer a clearer perception of the scale´s psychometric properties, indicating its positive and negative qualities. Our conclusions are in line with previously published works [6, 15, 16, 25, 37, 38]. The present evaluation should be of interest to the clinicians who administer the scale, and to the investigators who may wish to improve the available information. Exploring the CMS properties in different intervention types or developing variations of the scale, applicable to certain shoulder pathologies for example, could be possible future investigation lines.

## Conclusions

The CMS use is advisable for patients with subacromial pathology. As far as other shoulder conditions are concerned, the evidence suggests certain capacity in capturing changes over time, but the data were not conclusive. The obtained results do not justify the CMS as a gold standard in shoulder evaluation. Prospective studies set up to explore the psychometric properties of the scale, particularly for fractures, arthritis, instability and frozen shoulder are needed.

## Electronic supplementary material

Below is the link to the electronic supplementary material.

Online Appendix 1 (DOCX 14 KB)

Online Appendix 2 (DOCX 36 KB)

## Data Availability

The articles on which the standardized evaluations were based are presented in Online Appendix 2. Final standardized evaluations are presented in Table 2. Individual standardized evaluations are available from the corresponding author, upon reasonable request.
