# Supplementary material for: Constant–Murley Score: systematic review and standardized evaluation in different shoulder pathologies
Source: Qual Life Res. 2018 May 10;27(9):2217–26. doi: 10.1007/s11136-018-1875-7 (PMC6132990; doi:10.1007/s11136-018-1875-7)
Supplement: Supplementary file 1 — Online Appendix 1 (DOCX 14 KB) [file 11136_2018_1875_MOESM1_ESM.docx]

**Appendix 1 :** MEDLINE and EMBASE specific search strategies.

**Data search: 02/05/2014**

**A. MEDLINE**

| **Search term** |
| --- |
| 1.. (((((((constant[All Fields] AND score[All Fields]) OR (constant[All Fields] AND murley[All Fields])) OR (constant[All Fields] AND murley[All Fields] AND score[All Fields])) OR (constant[All Fields] AND ("shoulder"[MeSH Terms] OR "shoulder"[All Fields]))) OR (constant[All Fields] AND ("shoulder"[MeSH Terms] OR "shoulder"[All Fields]) AND score[All Fields])) OR constant-murley[All Fields]) OR (constant[All Fields] AND ("weights and measures"[MeSH Terms] OR ("weights"[All Fields] AND "measures"[All Fields]) OR "weights and measures"[All Fields] OR "scale"[All Fields]))) AND ("1986/01/01"[PDAT] : "2014/05/02"[PDAT]) |
| 2. ("shoulder"[MeSH Terms] OR "shoulder"[All Fields]) |
| 3. 1 AND 2 |

**B. EMBASE**

| **Search term** |
| --- |
| 1. 'Constant'/exp OR 'constant murley (score)'/exp OR 'constant and murley score'/exp OR 'constant score'/exp ' OR constant murley score'/exp OR 'constant shoulder'/exp OR 'constant shoulder score'/exp OR 'constant-murley'/exp OR 'constant-scale'/exp OR  constant shoulder score/de OR constant shoulder/de OR constant murley score/de OR constant murley/de OR constant score/de OR constant scale/de |
| 2. should* OR 'shoulder'/exp OR shoulder |
| 3. 1 AND 2 |
| 4. #3 AND [embase]/lim AND [1986-2014]/py |
