# Supplementary material for: Constant–Murley Score: systematic review and standardized evaluation in different shoulder pathologies
Source: Qual Life Res. 2018 May 10;27(9):2217–26. doi: 10.1007/s11136-018-1875-7 (PMC6132990; doi:10.1007/s11136-018-1875-7)
Supplement: Supplementary file 2 — Online Appendix 2 (DOCX 36 KB) [file 11136_2018_1875_MOESM2_ESM.docx]

**Appendix 2: Full list of evaluated articles**

**1. Subacromial pathology: 37 articles**

| 1 | Urvoy P, Boileau G, Berger M, Vanvelcenaher J, Schmidt D, Herlant M, et al. [Correlation and validation of different methods of evaluation of results after surgery of the rotator cuff. Plea for a standardized method]. Rev Chir Orthop Reparatrice Appar Mot 1991;77(3):171-8. |
| --- | --- |
| 2 | Gazielly DF, Gleyze P, Montagnon C. Functional and anatomical results after rotator cuff repair. Clin Orthop Relat Res 1994 Jul;(304):43-53. |
| 3 | Gazielly DF, Gleyze P, Montagnon C, Bruyere G, Prallet B. [Functional and anatomical results after surgical treatment of ruptures of the rotator cuff. 1: Preoperative functional and anatomical evaluation of ruptures of the rotator cuff]. Rev Chir Orthop Reparatrice Appar Mot 1995;81(1):8-16. |
| 4 | Boussagol B, Pelissier J, Herisson C, Simon L. [Clinical assessment of rotator cuff lesions: Comparative analysis of four scales]. Ann Readapt Med Phys 1996;39(2):79-88. |
| 5 | T'Jonck L, Lysens R, De SL, Bellemans J, Stoffelen D, Tirez B, et al. Open versus arthroscopic subacromial decompression: analysis of one-year results. Physiother Res Int 1997;2(2):46-61. |
| 6 | Gartsman GM, Brinker MR, Khan M. Early effectiveness of arthroscopic repair for full-thickness tears of the rotator cuff: an outcome analysis. J Bone Joint Surg Am 1998 Jan;80(1):33-40. |
| 7 | O'Connor DA, Chipchase LS, Tomlinson J, Krishnan J. Arthroscopic subacromial decompression: responsiveness of disease-specific and health-related quality of life outcome measures. Arthroscopy 1999 Nov;15(8):836-40. |
| 8 | Skutek M, Fremerey RW, Zeichen J, Bosch U. Outcome analysis following open rotator cuff repair. Early effectiveness validated using four different shoulder assessment scales. Arch Orthop Trauma Surg 2000;120(7-8):432-6. |
| 9 | Torrens C, Orient F, Vila F, Escalada F, Marín M. Análisis comparativo entre la escala de Constant y el cuestionario de salud SF-36 en pacientes con patología subacromial. Revista de Ortopedia y Traumatología 2000;44:447-51. |
| 10 | Dawson J, Hill G, Fitzpatrick R, Carr A. The benefits of using patient-based methods of assessment. Medium-term results of an observational study of shoulder surgery. J Bone Joint Surg Br 2001 Aug;83(6):877-82. |
| 11 | Skutek M, Zeichen J, Fremerey RW, Bosch U. [Outcome analysis after open reconstruction of rotator cuff ruptures. A comparative assessment of recent evaluation procedures]. Unfallchirurg 2001 Jun;104(6):480-7. |
| 12 | Dawson J, Hill G, Fitzpatrick R, Carr A. Comparison of clinical and patient-based measures to assess medium-term outcomes following shoulder surgery for disorders of the rotator cuff. Arthritis Rheum 2002 Oct 15;47(5):513-9. |
| 13 | Kirkley A, Alvarez C, Griffin S. The development and evaluation of a disease-specific quality-of-life questionnaire for disorders of the rotator cuff: The Western Ontario Rotator Cuff Index. Clin J Sport Med 2003 Mar;13(2):84-92. |
| 14 | Huber W, Hofstaetter JG, Hanslik-Schnabel B, Posch M, Wurnig C. The German version of the Oxford Shoulder Score--cross-cultural adaptation and validation. Arch Orthop Trauma Surg 2004 Oct;124(8):531-6. |
| 15 | Porcellini G, Paladini P, Campi F, Paganelli M. Arthroscopic treatment of calcifying tendinitis of the shoulder: clinical and ultrasonographic follow-up findings at two to five years. J Shoulder Elbow Surg 2004 Sep;13(5):503-8. |
| 16 | Romeo AA, Mazzocca A, Hang DW, Shott S, Bach BR, Jr. Shoulder scoring scales for the evaluation of rotator cuff repair. Clin Orthop Relat Res 2004 Oct;(427):107-14. |
| 17 | Holtby R, Razmjou H. Measurement properties of the Western Ontario rotator cuff outcome  measure: a preliminary report. J Shoulder Elbow Surg 2005 Sep;14(5):506-10. |
| 18 | Huber W, Hofstaetter JG, Hanslik-Schnabel B, Posch M, Wurnig C. [Translation and psychometric testing of the Rotator Cuff Quality-of-Life Measure (RC-QOL) for use in German-speaking regions]. Z Rheumatol 2005 Apr;64(3):188-97. |
| 19 | Huber W, Hofstaetter JG, Hanslik-Schnabel B, Posch M, Wurnig C. [Translation and psychometric testing of the Western Ontario Rotator Cuff Index (WORC) for use in Germany]. Z Orthop Ihre Grenzgeb 2005 Jul;143(4):453-60. |
| 20 | Wollmerstedt N, Bohm DT, Kirschner S, Kohler M, Konig A. [Evaluation of a simple test for shoulder function in patients with surgically treated rotator cuff injuries]. Z Orthop Ihre Grenzgeb 2005 Jul;143(4):468-74. |
| 21 | El O, Bircan C, Gulbahar S, Demiral Y, Sahin E, Baydar M, et al. The reliability and validity of the Turkish version of the Western Ontario Rotator Cuff Index. Rheumatol Int 2006 Oct;26(12):1101-8. |
| 22 | Livain T, Pichon H, Vermeulen J, Vaillant J, Saragaglia D, Poisson MF, et al. [Intra- and interobserver reproducibility of the French version of the Constant-Murley shoulder assessment during rehabilitation after rotator cuff surgery]. Rev Chir Orthop Reparatrice Appar Mot 2007 Apr;93(2):142-9. |
| 23 | Ghroubi S, Chaari M, Elleuch H, Guermazi M, Baklouti S, Elleuch MH. [Functional and quality of life outcome of non-operated rotator cuff tears]. Ann Readapt Med Phys 2008 Dec;51(9):714-21. |
| 24 | Gialanella B. [Disability and occupational therapy in patient with tear of the rotator cuff]. G Ital Med Lav Ergon 2008 Jan;30(1):78-82. |
| 25 | Razmjou H, Bean A, Macdermid JC, van O, V, Travers N, Holtby R. Convergent validity of the constant-murley outcome measure in patients with rotator cuff disease. Physiother Can 2008;60(1):72-9. |
| 26 | Tae SK, Rhee YG, Park TS, Lee KW, Park JY, Choi CH, et al. The development and validation of an appraisal method for rotator cuff disorders: the Korean Shoulder Scoring System. J Shoulder Elbow Surg 2009 Sep;18(5):689-96. |
| 27 | Goutallier D, Postel JM, Chevalier X, Beaudreuil J, Zilber S. Intermediate term functional outcome prediction following full thickness rotator cuff tear reparative or not reparative surgery. Orthop Traumatol Surg Res 2010 Nov;96(7):727-33. |
| 28 | Longo UG, Vasta S, Maffulli N, Denaro V. Scoring systems for the functional assessment of patients with rotator cuff pathology. Sports Med Arthrosc 2011 Sep;19(3):310-20. |
| 29 | Wessel RN, Lim TE, van MH, de Bie RA. Validation of the Western Ontario Rotator Cuff index in patients with arthroscopic rotator cuff repair: a study protocol. BMC Musculoskelet Disord 2011;12:64. |
| 30 | Chung SW, Park JS, Kim SH, Shin SH, Oh JH. Quality of life after arthroscopic rotator cuff repair: evaluation using SF-36 and an analysis of affecting clinical factors. Am J Sports Med 2012 Mar;40(3):631-9. |
| 31 | de Witte PB, Henseler JF, Nagels J, Vliet Vlieland TP, Nelissen RG. The Western Ontario rotator cuff index in rotator cuff disease patients: a comprehensive reliability and responsiveness validation study. Am J Sports Med 2012 Jul;40(7):1611-9. |
| 32 | Razmjou H, Stratford P, Holtby R. A shortened version of the Western ontario rotator cuff disability index: development and measurement properties. Physiother Can 2012;64(2):135-44. |
| 33 | Karas V, Hussey K, Romeo AR, Verma N, Cole BJ, Mather RC, III. Comparison of subjective and objective outcomes after rotator cuff repair. Arthroscopy 2013 Nov;29(11):1755-61. |
| 34 | Kukkonen J, Kauko T, Vahlberg T, Joukainen A, Aarimaa V. Investigating minimal clinically important difference for Constant score in patients undergoing rotator cuff surgery. J Shoulder Elbow Surg 2013 Dec;22(12):1650-5. |
| 35 | Wessel RN, Wolterbeek N, Fermont AJ, van MH, Sonneveld H, Griffin S, et al. The conceptually equivalent Dutch version of the Western Ontario Rotator Cuff Index (WORC)(c). BMC Musculoskelet Disord 2013;14:362. |
| 36 | Holmgren T, Oberg B, Adolfsson L, Bjornsson HH, Johansson K. Minimal important changes in the Constant-Murley score in patients with subacromial pain. J Shoulder Elbow Surg 2014 Aug;23(8):1083-90. |
| 37 | Moeller AD, Thorsen RR, Torabi TP, Bjoerkman AS, Christensen EH, Maribo T, et al. The Danish version of the modified Constant-Murley shoulder score: reliability, agreement, and construct validity. J Orthop Sports Phys Ther 2014 May;44(5):336-40. |

Additional articles from another pathology group

| - | Conboy VB, Morris RW, Kiss J, Carr AJ. An evaluation of the Constant-Murley shoulder assessment. J Bone Joint Surg Br 1996; 78(2):229-232. |
| --- | --- |
| - | Gilbart MK, Gerber C. Comparison of the subjective shoulder value and the Constant score. J Shoulder Elbow Surg 2007; 16(6):717-721. |

**2. Fractures: 7 articles**

| 1 | Tingart M, Bathis H, Lefering R, Bouillon B, Tiling T. [Constant Score and Neer Score. A comparison of score results and subjective patient satisfaction]. Unfallchirurg 2001 Nov;104(11):1048-54. |
| --- | --- |
| 2 | Baker P, Nanda R, Goodchild L, Finn P, Rangan A. A comparison of the Constant and Oxford shoulder scores in patients with conservatively treated proximal humeral fractures. J Shoulder Elbow Surg 2008 Jan;17(1):37-41. |
| 3 | Poeze M, Lenssen AF, Van Empel JM, Verbruggen JP. Conservative management of proximal humeral fractures: can poor functional outcome be related to standard transscapular radiographic evaluation? J Shoulder Elbow Surg 2010 Mar;19(2):273-81. |
| 4 | Slobogean GP, Slobogean BL. Measuring shoulder injury function: common scales and checklists. Injury 2011 Mar;42(3):248-52. |
| 5 | Urda A, Gonzalez A, Colino A, Lopiz Y, Garcia-Fernandez C, Marco F. Management of displaced surgical neck fractures of the humerus: health related quality of life, functional and radiographic results. Injury 2012 Dec;43 Suppl 2:S12-S19. |
| 6 | Inauen C, Platz A, Meier C, Zingg U, Rufibach K, Spross C, et al. Quality of life after osteosynthesis of fractures of the proximal humerus. J Orthop Trauma 2013 Apr;27(4):e74-e80. |
| 7 | van de Water AT, Shields N, Davidson M, Evans M, Taylor NF. Reliability and validity of shoulder function outcome measures in people with a proximal humeral fracture. Disabil Rehabil 2014;36(13):1072-9. |

## 3.Arthritis: 6 articles

| 1 | van den Ende CH, Rozing PM, Dijkmans BA, Verhoef JA, Voogt-van der Harst EM, Hazes JM. Assessment of shoulder function in rheumatoid arthritis. J Rheumatol 1996 Dec;23(12):2043-8. |
| --- | --- |
| 2 | Angst F, Pap G, Mannion AF, Herren DB, Aeschlimann A, Schwyzer HK, et al. Comprehensive assessment of clinical outcome and quality of life after total shoulder arthroplasty: usefulness and validity of subjective outcome measures. Arthritis Rheum 2004 Oct 15;51(5):819-28. |
| 3 | Angst F, Goldhahn J, Drerup S, Aeschlimann A, Schwyzer HK, Simmen BR. Responsiveness of six outcome assessment instruments in total shoulder arthroplasty. Arthritis Rheum 2008 Mar 15;59(3):391-8. |
| 4 | Christie A, Hagen KB, Mowinckel P, Dagfinrud H. Methodological properties of six shoulder disability measures in patients with rheumatic diseases referred for shoulder surgery. J Shoulder Elbow Surg 2009 Jan;18(1):89-95. |
| 5 | Christie A, Dagfinrud H, Garratt AM, Ringen OH, Hagen KB. Identification of shoulder-specific patient acceptable symptom state in patients with rheumatic diseases undergoing shoulder surgery. J Hand Ther 2011 Jan;24(1):53-60. |
| 6 | Rasmussen JV, Jakobsen J, Olsen BS, Brorson S. Translation and validation of the Western Ontario Osteoarthritis of the Shoulder (WOOS) index - the Danish version. Patient Relat Outcome Meas 2013;4:49-54. |
|  |  |

**Additional articles from another pathology group**

| - | Conboy VB, Morris RW, Kiss J, Carr AJ. An evaluation of the Constant-Murley shoulder assessment. J Bone Joint Surg Br 1996 Mar;78(2):229-32. |
| --- | --- |
| - | Gilbart MK, Gerber C. Comparison of the subjective shoulder value and the Constant score. J Shoulder Elbow Surg 2007 Nov;16(6):717-21. |

## 4.Instability: 5 articles

| 1 | Kirkley A, Griffin S, McLintock H, Ng L. The development and evaluation of a disease-specific quality of life measurement tool for shoulder instability. The Western Ontario Shoulder Instability Index (WOSI). Am J Sports Med 1998 Nov;26(6):764-72. |
| --- | --- |
| 2 | Dawson J, Fitzpatrick R, Carr A. The assessment of shoulder instability. The development and validation of a questionnaire. J Bone Joint Surg Br 1999 May;81(3):420-6. |
| 3 | Plancher KD, Lipnick SL. Analysis of evidence-based medicine for shoulder instability. Arthroscopy 2009 Aug;25(8):897-908. |
| 4 | Hofstaetter JG, Hanslik-Schnabel B, Hofstaetter SG, Wurnig C, Huber W. Cross-cultural adaptation and validation of the German version of the Western Ontario Shoulder Instability index. Arch Orthop Trauma Surg 2010 Jun;130(6):787-96. |
| 5 | Kemp KA, Sheps DM, Beaupre LA, Styles-Tripp F, Luciak-Corea C, Balyk R. An evaluation of the responsiveness and discriminant validity of shoulder questionnaires among patients receiving surgical correction of shoulder instability. ScientificWorldJournal 2012;2012:410125. |

**Additional articles from another pathology group**

| - | Gilbart MK, Gerber C. Comparison of the subjective shoulder value and the Constant score. J Shoulder Elbow Surg 2007 Nov;16(6):717-21. |
| --- | --- |

## 5.Frozen Shoulder: 1 article

| 1 | Othman A, Taylor G. Is the constant score reliable in assessing patients with frozen shoulder? 60 shoulders scored 3 years after manipulation under anaesthesia. Acta Orthop Scand 2004 Feb;75(1):114-6. |
| --- | --- |

## 6.Various Pathologies: 29 articles

| 1 | Conboy VB, Morris RW, Kiss J, Carr AJ. An evaluation of the Constant-Murley shoulder assessment. J Bone Joint Surg Br 1996 Mar;78(2):229-32. | |  |
| --- | --- | --- | --- |
| 2 | Dawson J, Fitzpatrick R, Carr A. Questionnaire on the perceptions of patients about shoulder surgery. J Bone Joint Surg Br 1996 Jul;78(4):593-600. | |  |
| 3 | Kohn D, Geyer M. The subjective shoulder rating system. Arch Orthop Trauma Surg 1997;116(6-7):324-8. | |  |
| 4 | Bankes MJ, Crossman JE, Emery RJ. A standard method of shoulder strength measurement for the Constant score with a spring balance. J Shoulder Elbow Surg 1998 Mar;7(2):116-21. | |  |
| 5 | Taylor RJ, Chepeha JC, Teknos TN, Bradford CR, Sharma PK, Terrell JE, et al. Development and validation of the neck dissection impairment index: a quality of life measure. Arch Otolaryngol Head Neck Surg 2002 Jan;128(1):44-9. | |  |
| 6 | Cook KF, Roddey TS, Olson SL, Gartsman GM, Valenzuela FF, Hanten WP. Reliability by surgical status of self-reported outcomes in patients who have shoulder pathologies. J Orthop Sports Phys Ther 2002 Jul;32(7):336-46. | |  |
| 7 | Boehm D, Wollmerstedt N, Doesch M, Handwerker M, Mehling E, Gohlke F. [Development of a questionnaire based on the Constant-Murley-Score for self-evaluation of shoulder function by patients]. Unfallchirurg 2004 May;107(5):397-402. | |  |
| 8 | Placzek JD, Lukens SC, Badalanmenti S, Roubal PJ, Freeman DC, Walleman KM, et al. Shoulder outcome measures: a comparison of 6 functional tests. Am J Sports Med 2004 Jul;32(5):1270-7. | |  |
| 9 | Fialka C, Oberleitner G, Stampfl P, Brannath W, Hexel M, Vecsei V. Modification of the Constant-Murley shoulder score-introduction of the individual relative Constant score Individual shoulder assessment. Injury 2005 Oct;36(10):1159-65. | |  |
| 10 | Leggin BG, Michener LA, Shaffer MA, Brenneman SK, Iannotti JP, Williams GR, Jr. The Penn shoulder score: reliability and validity. J Orthop Sports Phys Ther 2006 Mar;36(3):138-51. | |  |
| 11 | Gilbart MK, Gerber C. Comparison of the subjective shoulder value and the Constant score. J Shoulder Elbow Surg 2007 Nov;16(6):717-21. | |  |
| 12 | Fayad F, Lefevre-Colau MM, Mace Y, Fermanian J, Mayoux-Benhamou A, Roren A, et al. Validation of the French version of the Disability of the Arm, Shoulder and Hand questionnaire (F-DASH). Joint Bone Spine 2008 Mar;75(2):195-200. | |  |
| 13 | Rocourt MH, Radlinger L, Kalberer F, Sanavi S, Schmid NS, Leunig M, et al. Evaluation of intratester and intertester reliability of the Constant-Murley shoulder assessment. J Shoulder Elbow Surg 2008 Mar;17(2):364-9. | |  |
| 14 | Ozsahin M, Akgun K, Aktas I, Kurtais Y. Adaptation of the Shoulder Disability Questionnaire to the Turkish population, its reliability and validity. Int J Rehabil Res 2008 Sep;31(3):241-5. | |  |
| 15 | Fayad F, Lefevre-Colau MM, Gautheron V, Mace Y, Fermanian J, Mayoux-Benhamou A, et al. Reliability, validity and responsiveness of the French version of the questionnaire Quick Disability of the Arm, Shoulder and Hand in shoulder disorders. Man Ther 2009 Apr;14(2):206-12. | |  |
| 16 | Oh JH, Jo KH, Kim WS, Gong HS, Han SG, Kim YH. Comparative evaluation of the measurement properties of various shoulder outcome instruments. Am J Sports Med 2009 Jun;37(6):1161-8. | |  |
| 17 | Clement ND, Fuller M, Colling RC, Stirrat AN. Assessment of shoulder function using the coronal plane angle. Int J Shoulder Surg 2009 Oct;3(4):90-3. | |  |
| 18 | Berendes T, Pilot P, Willems J, Verburg H, te SR. Validation of the Dutch version of the Oxford Shoulder Score. J Shoulder Elbow Surg 2010 Sep;19(6):829-36. | |  |
| 19 | Murena L, Vulcano E, D'Angelo F, Monti M, Cherubino P. Italian cross-cultural adaptation and validation of the Oxford Shoulder Score. J Shoulder Elbow Surg 2010 Apr;19(3):335-41. | |  |
| 20 | Dupeyron A, Gelis A, Sablayrolles P, Bousquet PJ, Julia M, Herisson C, et al. Heterogeneous assessment of shoulder disorders: validation of the Standardized Index of Shoulder Function. J Rehabil Med 2010 Nov;42(10):967-72. | |  |
| 21 | Blonna D, Scelsi M, Marini E, Bellato E, Tellini A, Rossi R, et al. Can we improve the reliability of the Constant-Murley score? J Shoulder Elbow Surg 2012 Jan;21(1):4-12. | |  |
| 22 | Frich LH, Noergaard PM, Brorson S. Validation of the Danish version of Oxford Shoulder Score. Dan Med Bull 2011 Nov;58(11):A4335. | |  |
| 23 | Noorani AM, Roberts DJ, Malone AA, Waters TS, Jaggi A, Lambert SM, et al. Validation of the Stanmore percentage of normal shoulder assessment. Int J Shoulder Surg 2012 Jan;6(1):9-14. | |  |
| 24 | Bafus BT, Hughes RE, Miller BS, Carpenter JE. Evaluation of utility in shoulder pathology: Correlating the American Shoulder and Elbow Surgeons and Constant scores to the EuroQoL. World J Orthop 2012 Mar 18;3(3):20-4. | |  |
| 25 | | van Kampen DA, van Beers LW, Scholtes VA, Terwee CB, Willems WJ. Validation  of the Dutch version of the Simple Shoulder Test. J Shoulder Elbow Surg 2012  Jun;21(6):808-14. | |
| 26 | Roh YH, Noh JH, Oh JH, Baek GH, Gong HS. To what degree do shoulder outcome instruments reflect patients' psychologic distress? Clin Orthop Relat Res 2012 Dec;470(12):3470-7. | |  |
| 27 | Schmidutz F, Beirer M, Braunstein V, Bogner V, Wiedemann E, Biberthaler P. The Munich Shoulder Questionnaire (MSQ): development and validation of an effective patient-reported tool for outcome measurement and patient safety in shoulder surgery. Patient Saf Surg 2012;6(1):9. | |  |
| 28 | Ge Y, Chen S, Chen J, Hua Y, Li Y. The development and evaluation of a new shoulder scoring system based on the view of patients and physicians: the Fudan University shoulder score. Arthroscopy 2013 Apr;29(4):613-22. | |  |
| 29 | Levy O, Haddo O, Massoud S, Mullett H, Atoun E. A patient-derived Constant-Murley score is comparable to a clinician-derived score. Clin Orthop Relat Res 2014 Jan;472(1):294-303. | |  |

## 7. Healthy Subjects: 9 articles

| 1 | Brinker MR, Cuomo JS, Popham GJ, O'Connor DP, Barrack RL. An examination of bias in shoulder scoring instruments among healthy collegiate and recreational athletes. *J Shoulder Elbow Surg* 2002; 11(5):463-469. |
| --- | --- |
| 2 | Grassi FA, Tajana MS. The normalization of data in the Constant-Murley score for the shoulder. A study conducted on 563 healthy subjects. *Chir Organi Mov* 2003; 88(1):65-73. |
| 3 | Thomas M, Dieball O, Busse M. [Normal values of the shoulder strength in dependency on age and gender--comparison with the constant, UCLA, ASES scores and SF36 health survey]. *Z Orthop Ihre Grenzgeb* 2003; 141(2):160-170. |
| 4 | Johansson KM, Adolfsson LE. Intraobserver and interobserver reliability for the strength test in the Constant-Murley shoulder assessment. *J Shoulder Elbow Surg* 2005; 14(3):273-278. |
| 5 | Katolik LI, Romeo AA, Cole BJ, Verma NN, Hayden JK, Bach BR. Normalization of the Constant score. *J Shoulder Elbow Surg* 2005; 14(3):279-285. |
| 6 | Yian EH, Ramappa AJ, Arneberg O, Gerber C. The Constant score in normal shoulders. *J Shoulder Elbow Surg* 2005; 14(2):128-133. |
| 7 | Walton MJ, Walton JC, Honorez LA, Harding VF, Wallace WA. A comparison of methods for shoulder strength assessment and analysis of Constant score change in patients aged over fifty years in the United Kingdom. *J Shoulder Elbow Surg* 2007; 16(3):285-289. |
| 8 | Tavakkolizadeh A, Ghassemi A, Colegate-Stone T, Latif A, Sinha J. Gender-specific Constant score correction for age. *Knee Surg Sports Traumatol Arthrosc* 2009; 17(5):529-533. |
| 9 | Hirschmann MT, Wind B, Amsler F, Gross T. Reliability of shoulder abduction strength measure for the Constant-Murley score. *Clin Orthop Relat Res* 2010; 468(6):1565-1571. |

**Additional articles from another pathology group**

| - | Bankes MJ, Crossman JE, Emery RJ. A standard method of shoulder strength measurement for the Constant score with a spring balance. *J Shoulder Elbow Surg* 1998; 7(2):116-121. |
| --- | --- |
| - | Fialka C, Oberleitner G, Stampfl P, Brannath W, Hexel M, Vecsei V. Modification of the Constant-Murley shoulder score-introduction of the individual relative Constant score Individual shoulder assessment. *Injury* 2005; 36(10):1159-1165. |

**Concept and measurement model attribute: 2 articles**

| 1 | Constant CR, Murley AH. [A clinical method of functional assessment of the shoulder.](http://www.ncbi.nlm.nih.gov/pubmed/3791738) *Clin Orthop Relat Res. 1987*;(214):160-4. |
| --- | --- |
| 2 | Constant CR, Gerber C, Emery RJ, Søjbjerg JO, Gohlke F, Boileau P. [A review of the Constant score: modifications and guidelines for its use.](http://www.ncbi.nlm.nih.gov/pubmed/18218327) *J Shoulder Elbow Surg. 2008 Mar-Apr;17(2):355-61.* |
